# Interferons in HIV-1 infection: mechanisms, antiviral potentials, and therapeutic challenges

**DOI:** 10.3389/fimmu.2025.1736658

**Published:** 2025-12-18

**Authors:** Luying Zhu, Jiahao Ji, Jing Xiao, Fuchun Wang, Jiaqi Yu, Yanmin Liu, Yang Zhang, Hao Wu, Bin Su, Xiaofan Lu, Tong Zhang

**Affiliations:** 1 Clinical and Research Center for Infectious Diseases, Beijing Youan Hospital, Beijing, China; 2 Beijing Key Laboratory for HIV/AIDS Research, Beijing Youan Hospital, Capital Medical University, Beijing, China; 3 Second Department of Liver Disease Center, Beijing Youan Hospital, Capital Medical University, Beijing, China

**Keywords:** antiviral mechanisms, HIV-1 infection, IFN-α therapy, immunomodulation, interferons

## Abstract

Type I interferons (IFNs), particularly IFN-α, occupy a central paradox in HIV-1 infection: they provide an essential early antiviral barrier that limits initial dissemination, yet their sustained activation contributes to chronic immune activation, CD4^+^ T-cell dysfunction, and incomplete viral control. This duality—protective in acute infection but pathogenic during chronic disease—remains a major unresolved challenge for interferon-based therapeutic strategies in HIV-1. Recent advances in ISG functional profiling, IFN-α subtype–specific antiviral potency, and the development of targeted innate-pathway modulators (e.g., STING-selective agonists, ncRNA regulators, TLR7 activators) have renewed interest in reevaluating interferon-centered approaches. These developments make it timely to reassess whether IFN-α can be safely and effectively integrated into modern HIV-1 therapeutic concepts, particularly in early-infection windows or in rationally designed combination regimens. In this review, we synthesize current knowledge of interferon-mediated restriction mechanisms, the hierarchy of key antiviral ISGs (e.g., APOBEC3G, MX2, BST2), and HIV-1 evasion of the JAK–STAT and cGAS–STING pathways. We further analyze how dose, timing, and IFN-α subtype contribute to divergent antiviral versus inflammatory outcomes across different stages of infection. Emerging precision strategies that modulate interferon signaling without triggering systemic inflammation offer promising translational directions. Balancing antiviral efficacy with immune homeostasis will be essential for developing next-generation interferon-based interventions aimed at durable control or functional cure of HIV-1 infection.

## 1 Fundamental roles of interferon signaling: overview of antiviral mechanisms of type I, II, and III IFNs

Interferons (IFNs) constitute a multifunctional cytokine system that integrates innate and adaptive immunity and shapes host–virus interactions across diverse infections. To provide a conceptual foundation for understanding their relevance in HIV-1 infection, this section begins with a comparative overview of the three IFN families—type I, type II, and type III—highlighting their cellular sources, receptor distribution, and immunological consequences before addressing their clinical applications.

Type I IFNs form the most expansive IFN family and include 13 IFN-α subtypes as well as IFN-β, IFN-ϵ, IFN-κ, and IFN-ω. All signal through the widely expressed IFNAR1/IFNAR2 receptor complex, activating the JAK–STAT pathway to form the ISGF3 transcription factor and induce hundreds of ISGs (1).These ISGs—such as OAS, PKR, and MX proteins—establish a potent antiviral state, with MX2 serving as a prominent HIV-1 restriction factor by blocking capsid-dependent nuclear import (2, 3). Because IFNAR is nearly ubiquitous, type I IFNs exert broad systemic effects across hematopoietic and non-hematopoietic tissues. Defects in IFN-I production or anti–IFN autoantibodies are linked to severe viral diseases such as COVID-19, underscoring their essential antiviral function (4).However, this same breadth of activity underlies their pathogenic potential. Insights from autoimmune diseases—including systemic lupus erythematosus, rheumatoid arthritis, and primary Sjögren’s syndrome—demonstrate how chronic, dysregulated IFN-I signaling drives aberrant immune activation, antigen-presenting cell dysregulation, and T-cell dysfunction (5–9). These mechanisms provide a conceptual bridge to HIV-1: chronic HIV infection similarly features persistent yet sub-protective IFN-I activity that fuels immune activation, inflammatory cytokine production, and epithelial barrier perturbation.Thus, autoimmune disease is not an irrelevant detour but an instructive model of how prolonged IFN-I exposure becomes maladaptive during persistent viral infection.

Whereas type I IFNs are pleiotropic, type II IFN (IFN-γ) has a narrower functional spectrum, produced by multiple lymphocyte subsets including αβCD4^+^ and αβCD8^+^ T cells, γδ T cells, invariant natural killer T (iNKT) cells, mucosa-associated invariant T cells, and natural killer (NK) cells (10). In contrast to the broad antiviral activity of IFN-I, IFN-γ functions as a macrophage-activating factor (MAF) with a unique ability to promote clearance of intracellular pathogens—including bacteria, fungi, protozoa, and certain viruses—by macrophages, as demonstrated in studies of leprosy treatment in the mid-1980s (11). During early HIV-1 infection, IFN-γ contributes to antiviral defense; however, persistently elevated IFN-γ in chronic disease correlates with systemic immune activation and markers of disease progression rather than durable virological control. This distinction highlights that different IFN families exert phase-specific and sometimes maladaptive effects across the course of HIV infection.

Type III IFNs (IFN-λ1–4) signal through a receptor complex (IL-28Rα/IL-10Rβ) whose expression is largely restricted to epithelial cells of the gastrointestinal, respiratory, and reproductive tracts (12–14), as well as select immune cells such as dendritic cells and neutrophils (15–17). Although IFN-λ activates the same ISGF3 pathway as type I IFNs, its epithelial-restricted receptor distribution confers potent mucosal antiviral activity with substantially reduced systemic inflammation. This profile is particularly relevant to HIV-1, as the gastrointestinal mucosa—especially the gut-associated lymphoid tissue (GALT)—is among the earliest and most profoundly affected immune compartments, undergoing rapid memory CD4^+^ T-cell depletion and barrier dysfunction during acute infection (18). The biological properties of IFN-λ therefore provide a theoretical advantage for targeted antiviral activity in mucosal tissues while minimizing systemic immune activation.

After outlining endogenous IFN biology, we next address their clinical application. Recombinant IFN-α2a/α2b and IFN-β—biosynthetic analogues of leukocyte- and fibroblast-derived IFNs—are widely used in HBV (19), HCV (20), and HEV infections (21, 22). In HIV-1 and SIV models, exogenous IFN-α can reduce viral replication during early infection (23) and transiently decrease HIV-1 viremia in humans (24–26). IFN-γ has also been explored therapeutically, particularly in mycobacterial infections and selected immunodeficiency disorders (27–29), though its utility in HIV remains limited due to its association with chronic immune activation during established disease. Importantly, although IFN-λ signals through an epithelial-restricted receptor and generally exhibits a more favorable inflammatory and hematologic safety profile than IFN-α, its discovery and clinical development lagged far behind type I IFNs. Available hepatitis C trials show that pegylated IFN-λ can achieve antiviral responses comparable to pegylated IFN-α but with a more gradual and tissue-restricted induction of ISGs, rather than clearly superior or faster viral clearance (30). Together with the rapid displacement of interferon-based regimens by direct-acting antivirals, these features have limited the advancement of IFN-λ relative to IFN-α in regulatory approval and in HIV-focused clinical research.

Together, these comparative features of type I, II, and III IFNs establish the biological and clinical context for understanding how HIV-1 senses, responds to, evades, and potentially can be targeted through interferon-mediated pathways.

## 2 IFN-I signaling in HIV-1 infection: dynamics, molecular mechanisms, and immune evasion

### 2.1 Dynamics and biological characteristics of IFN-I signaling following HIV-1 infection

Following HIV-1 entry, the innate immune system rapidly detects viral nucleic acids and mounts a robust IFN-I response. In the early phase of infection, plasmacytoid dendritic cells (pDCs) serve as the predominant IFN-I producers, sensing viral RNA through Toll-like receptor 7/8 (TLR7/8) and activating interferon regulatory factor (IRF)–mediated pathways to secrete large amounts of IFN-α and IFN-β (31, 32). This early IFN-I burst induces restriction factors such as APOBEC3G, TRIM5α, and SAMHD1, which limit replication and dissemination (33, 34). The magnitude of this early response represents one of the host’s most critical nonspecific antiviral defenses, transiently suppressing viral spread and reducing the peak viremia in acute infection (35).

However, as infection progresses, HIV-1 evolves mechanisms that blunt IFN efficacy—by selecting IFN-resistant variants, expressing viral proteins (Vif, Vpu, Nef) that antagonize restriction factors, and suppressing innate signaling (33, 36). Clinical and animal studies demonstrate that although the acute-phase IFN-I peak correlates with reduced peak viremia, this protective effect is rarely sustained over time (37, 38).

In the chronic phase of HIV-1 infection, IFN-I levels decline substantially from their acute-phase peak but typically persist at a low-grade activation state (38, 39). This low-grade, persistent IFN-I tone is typically insufficient to control viral replication but contributes to chronic immune activation and inflammation. It is associated with sustained expression of T-cell activation markers (HLA-DR, CD38), upregulation of inhibitory receptors such as PD-1 and TIM-3, and increased susceptibility to apoptosis of bystander CD4^+^ T cells (38–41). Abortive infection of CD4^+^ T cells in lymphoid tissue further amplifies inflammatory death pathways, linking persistent innate sensing and IFN-I exposure to progressive immune depletion (40, 41).

**Figure 1:**
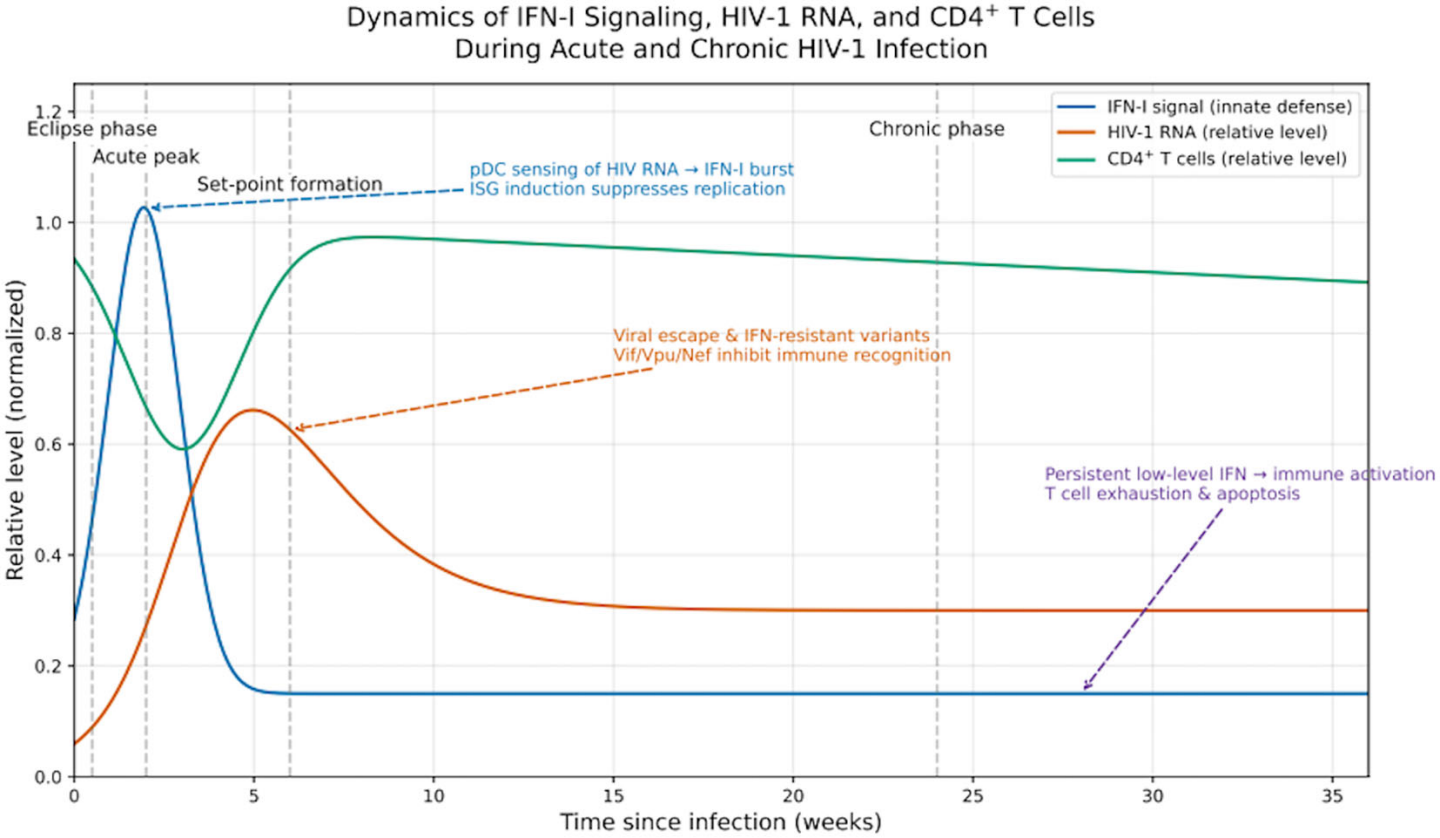
Dynamics of IFN-I signaling, plasma HIV-1 RNA, and CD4^+^ T-cell levels during acute and chronic HIV-1 infection. This schematic illustrates the temporal interplay among IFN-I responses, plasma viremia, and CD4^+^ T-cell dynamics after HIV-1 infection. IFN-I rises rapidly during the eclipse and acute phases (0–4 weeks), peaks with maximal viremia, and declines as adaptive immunity develops. HIV-1 RNA reaches a peak before falling to a set-point (4–8 weeks), whereas CD4^+^ T cells drop sharply, partially recover as IFN-I subsides, and gradually decline again during chronic infection (8w). Shaded areas mark Eclipse (0-2w), Acute Peak (2-4w), Set-point (4-8w), and Chronic (8w) phases. The overlap between IFN-I decline and CD4^+^ T-cell recovery is illustrative, not causal. Normalization: IFN-I and HIV-1 RNA curves are normalized to their acute-phase peaks (set = 1.0), and the CD4^+^ T-cell curve is normalized to the pre-infection baseline (set = 1.0). Values indicate relative, not absolute, levels. Source: Conceptual trends summarized from representative studies on acute and hyperacute HIV-1 infection and immune dynamics (43–47).

Collectively, IFN-I signaling during HIV infection exhibits a double-edged sword profile: a beneficial antiviral surge in the acute phase, contrasted with maladaptive chronic activation that perpetuates inflammation and T-cell depletion (42). **Figure 1** illustrates the temporal dynamics of IFN-I levels, plasma viral load, and CD4^+^ T-cell counts across the course of infection, highlighting the distinct biological impacts of IFN-I in different disease stages.

While pDCs are the primary source of the acute IFN-I burst in HIV-1 infection, evidence indicates that during the chronic phase IFN-I production is maintained at low but persistent levels by other cell types. Emerging data show that tissue-resident macrophages, particularly in gut-associated lymphoid tissue and lymph-node stromal compartments, can harbor latent HIV-1 infection while expressing IFN-stimulated gene signatures and low-grade IFN production (48). Residual pDCs also contribute, though their frequency declines in chronic infection (49). The sustained IFN-I tone appears not merely a passive remnant of acute activation, but may reflect qualitative changes in innate cell programming: chronic inflammatory cues may rewire macrophage and dendritic-cell responsiveness, driving a self-amplifying feedback loop of tonic ISG induction and immune activation rather than effective viral clearance.

Building upon the dynamic features and dual-phase effects of IFN-I signaling described above, the following section delineates the molecular pathways by which HIV-1 induces IFN-I production and the subsequent immunological consequences. This mechanistic insight provides the foundation for understanding both the antiviral and immunomodulatory roles of IFN-I in HIV-1 infection.

### 2.2 Molecular mechanisms and immunological roles of HIV-1–induced type I interferon responses

Upon HIV-1 infection, the viral genomic RNA undergoes reverse transcription to generate cDNA, which integrates into the host genome and contributes to the establishment of a latent viral reservoir. During this process, innate immune cells detect viral nucleic acids through multiple pattern-recognition receptors (PRRs) and cytosolic nucleic-acid sensors. In pDCs, HIV-1 RNA and immune complexes are sensed primarily via endosomal TLR7/9, leading to MyD88-dependent activation of IRF7 and robust production of IFN-α subtype (32). In parallel, in myeloid dendritic cells, macrophages, and resting CD4^+^ T cells, HIV-1-derived cDNA is recognized by the interferon-inducible DNA sensors IFI16 and cyclic GMP–AMP synthase (cGAS), which catalyzes the synthesis of cGAMP to activate the stimulator of interferon genes (STING) pathway. Activated STING recruits and activates TANK-binding kinase 1 (TBK1), leading to phosphorylation and dimerization of IRF3, which then translocates to the nucleus to drive transcription of IFN-β and early IFN-α subtypes (50–52).

In the acute phase, IFN-α is the dominant circulating IFN-I subtype and exerts both direct antiviral and immunomodulatory effects. It can inhibit HIV-1 replication at multiple stages of the viral life cycle, particularly before and immediately after integration, by inducing a spectrum of ISGs in primary CD4^+^ T cells and macrophages (1, 23, 53, 54) As an immunotherapeutic cytokine, IFN-α enhances innate cell function and transitions to adaptive responses by promoting CD8^+^ cytotoxicity and maintaining Th1 balance. Administration of IFN-α increases CD8^+^ T-cell activation and reduces plasma HIV RNA (55) and augments NK-cell degranulation and suppressive capacity against HIV-infected targets (56). Short-course IFN-α exposure *in vivo* reduces plasma HIV-1 RNA and upregulates ISG expression in peripheral blood, consistent with its role as a potent early-phase antiviral mediator (54–56).

Beyond direct antiviral effects, IFN-I signaling plays a critical role in linking innate detection to adaptive immunity. Type I IFNs promote maturation of conventional dendritic cells (cDCs) (up-regulation of CD80/86, MHC I/II) and enhance their ability to cross-present cell-associated viral antigens to CD8^+^ T cells. In viral infections including HIV-1, this IFN-driven DC activation supports the priming of virus-specific CD8^+^ T-cell responses and contributes to reservoir limitation (57, 58). Incorporating this role strengthens the mechanistic link between early IFN-I responses and durable adaptive immunity.

### 2.3 Role of IFN-α in suppressing HIV replication and limiting viral reservoir formation

Type I IFNs, particularly IFN-α, restrict HIV-1 replication through coordinated induction of ISGs that act at distinct stages of the viral life cycle. In primary macrophages and CD4^+^ T cells, IFN-α reduces the accumulation of HIV-1 cDNA and suppresses early replication through proteasome-dependent mechanisms (53), In resting CD4^+^ T cells, IFN-α potently enhances the antiviral activity of APOBEC3G, promoting G→A hypermutation in nascent viral DNA and thereby limiting residual replication within cells that contribute to the latent reservoir (59). *In vivo* studies further demonstrate that IFN-α treatment can lower HIV-1 viral load while upregulating the expression of restriction factors such as APOBEC3G/3F and BST-2/tetherin (54).

IFN-α-induced ISGs restrict HIV-1 replication at multiple checkpoints throughout the viral life cycle. (1) During viral entry and fusion, IFITM3 is strongly up-regulated and localizes to plasma and endosomal membranes, altering membrane fluidity and blocking fusion between viral and host membranes, thereby reducing entry efficiency (60). (2) During reverse transcription, SAMHD1 depletes intracellular dNTP pools in resting CD4^+^ T cells and macrophages, while CMPK2 maintains nucleotide balance under interferon signaling; together, these effects limit viral cDNA synthesis and halt reverse transcription (61, 62). (3) In the nuclear import or pre-integration stage, MX2 (MxB) acts post-reverse transcription but prior to integration by localizing to the nuclear envelope and interacting with nucleoporins (NUP214, TNPO1) to block nuclear entry of the pre-integration complex (63). In contrast, TRIM5α acts at an earlier post-entry step by recognizing the HIV-1 capsid, promoting its proteasomal degradation and preventing reverse transcription (64). (4) Following integration, APOBEC3G and APOBEC3F are packaged into virions, where they introduce G→A hypermutations that corrupt proviral genomes and reduce replication competence (59). (5) During virion assembly, ISG15 conjugates to viral and host budding factors such as Gag, hindering membrane fission and virion release, thereby reducing HIV-1 infectivity (65). (6) At the budding and release stage, BST-2/tetherin, highly expressed on activated CD4^+^ T cells and in lymphoid/mucosal tissues, anchors nascent virions at the cell surface, physically preventing virus release and cell-to-cell dissemination (66). Recent multi-omics analyses continue to expand the catalogue of cell-intrinsic effectors that contribute to IFN-I–mediated restriction of HIV-1 in primary CD4^+^ T cells (67). **Table 1** summarizes the major ISGs and their anti-HIV-1 mechanisms.

**Table 1:** Major IFN-α–induced ISGs and their mechanisms of HIV-1 restriction.

ISG/fost factor	Replication stage	Mechanism of action	Reference
IFITM3	Entry/Fusion	Alters membrane fluidity, blocks viral fusion	(60)
SAMHD1	Reverse Transcription	Depletes dNTP pools, blocks reverse transcription	(34, 61)
CMPK2	Reverse Transcription	Maintains UTP/CTP balance; knockout restores replication	(62, 68)
TRIM5α	Reverse Transcription (early)	Recognizes capsid, triggers proteasomal degradation	(64)
MX2 (MxB)	Nuclear Import	Blocks nuclear entry via nucleoporin interaction	(3, 63)
APOBEC3G/F	Post-integration	Induces G→A hypermutation, disrupts proviral genomes	(54, 59)
ISG15	Assembly	ISGylation of Gag/Env disrupts virion maturation	(65)
BST2 (Tetherin)	Budding/Release	Anchors virions to cell surface, inhibits release	(54, 66)

ISG, interferon-stimulated gene; IFN-α, interferon-alpha; dNTP, deoxynucleoside triphosphate; UTP, uridine triphosphate; CTP, cytidine triphosphate; MxB (MX2), myxovirus resistance protein B; G→A hypermutation, guanine-to-adenine base-editing induced by APOBEC3 family; ISGylation, post-translational modification by ISG15; Tetherin (BST2), bone-marrow stromal antigen 2; Nucleoporin, nuclear-pore complex protein involved in viral nuclear entry; Capsid, viral core protein shell; Virion, mature infectious viral particle.

This table summarizes representative ISGs that mediate antiviral restriction of HIV-1 across different replication stages. Each listed factor has been experimentally shown to limit infection through distinct molecular mechanisms, including inhibition of viral entry, reverse transcription, nuclear import, assembly, or budding.

Collectively, these IFN-α-induced antiviral mechanisms act in a temporally ordered cascade—from viral entry to release—to minimize productive infection and constrain the establishment of the latent reservoir. Despite the robust IFN-induced blockade observed *in vitro*, clinical efficacy *in vivo* remains limited, reflecting coordinated viral countermeasures (e.g., Vpu, Nef, capsid shielding), tissue-level constraints, and immune exhaustion driven by chronic interferon exposure. Together, these processes uncouple ISG induction from durable viral control in patients.

### 2.4 HIV immune evasion: targeting interferon signaling

HIV-1 employs temporally ordered immune-evasion strategies that align with distinct stages of its replication cycle, enabling the virus to circumvent IFN-I sensing, restrictor induction, and downstream effector pathways.

#### 2.4 .1Early phase: capsid-based evasion of nucleic-acid sensing

Immediately after entry, the HIV-1 capsid recruits host cofactors such as cyclophilin A and CPSF6 to stabilize its lattice and shield reverse-transcribed DNA from cytosolic sensors including cGAS and IFI16 (69).This capsid-cofactor “cloak” constitutes the earliest evasion layer, preventing STING–TBK1–IRF3 activation and blunting the initial IFN-I burst.

#### 2.4.2 Reverse-transcription stage: antagonism of intrinsic restriction factors

During reverse transcription, HIV-1 counters key ISGs: Vif mediates APOBEC3G/3F degradation, and Vpr modulates nucleotide metabolism to reduce SAMHD1-mediated restriction (33, 54, 62). These steps secure efficient synthesis of viral cDNA and formation of the pre-integration complex.

#### 2.4.3 Integration and early post-integration phase: chromatin remodeling and suppression of IFN-responsive loci

Following integration, HIV-1 shapes a chromatin environment that favors proviral persistence. Viral proteins can recruit HDACs and promote repressive marks such as H3K27me3 at interferon-regulated promoters (70, 71), while chronic IFN exposure further reinforces a transcriptionally restrained, latency-compatible state (72, 73). These epigenetic changes weaken IFN-inducible antiviral programs and stabilize the reservoir.

#### 2.4.4 Late phase: evasion of ISG-mediated suppression of virion release and infectivity

At the assembly and release stages, HIV-1 directly antagonizes ISG effector functions: Vpu counteracts BST-2/tetherin to permit virion release (74), and Nef inhibits SERINC5 to enhance the infectivity of progeny particles (75). These mechanisms operate downstream of reverse transcription and ensure productive viral egress even in an ISG-rich environment.

#### 2.4.5 Cross-phase regulation: epigenetic and ncRNA-mediated tuning of interferon pathways

Beyond stage-specific evasion events, HIV-1 also exploits cross-phase regulatory mechanisms that persist throughout the viral life cycle. Epigenetic modifications play a central role in this process. Histone modifications, particularly HDAC recruitment and PRC2-mediated H3K27 trimethylation, establish a transcriptionally repressive chromatin environment at the HIV-1 long terminal repeat (LTR) and interferon-responsive genes, thereby favoring latency rather than effective immune clearance (76–78). These changes are driven both by viral proteins and by prolonged exposure to inflammatory and interferon-rich conditions. Long non-coding RNAs further modulate chromatin structure and gene expression, adding an additional regulatory layer to innate immune programs (79).

In parallel, microRNAs regulate interferon signaling at the post-transcriptional level. Among them, miR-146a functions as a key negative-feedback regulator of innate immunity. HIV-1 infection induces miR-146a expression, which targets IRAK1 and TRAF6, reduces NF-κB activation, and attenuates interferon-stimulated gene expression (80, 81). Elevated miR-146a levels in chronic HIV-1 infection correlate with immune cell exhaustion and impaired antiviral responses (81). Together, these epigenetic and ncRNA-mediated mechanisms do not operate at a single replication stage, but provide sustained suppression of interferon pathways across acute and chronic phases of infection, thereby facilitating immune evasion and viral persistence.

Together, these analyses illustrate that IFN-I–driven antiviral restriction and HIV-1 immune evasion do not culminate in a unilateral “victory,” but instead establish a dynamic and pathologic host–virus equilibrium. IFN-I responses dominate briefly during acute infection, limiting early viral dissemination, yet HIV-1 rapidly acquires multilayered escape strategies that enable persistent replication and reservoir formation. Conversely, these escape adaptations incur fitness constraints on the virus, while the host maintains a chronically activated but sub-protective IFN-I milieu that restricts viral expansion without achieving clearance. This reciprocal adjustment between antiviral pressure and viral countermeasures forms a long-lasting, inflammation-prone steady state that underlies the clinical transition from acute containment to chronic immune dysregulation.

## 3 IFN-α in HIV-1 therapy: antiviral potential and immunological risks

### 3.1 Subtype specificity and clinical rationale for IFN-α2b

Although all 13 IFN-α subtypes signal through the shared IFNAR1/2-JAK-STAT/ISGF3 axis, they exhibit distinct biological profiles in antiviral potency and immune modulation. Comparative analyses consistently identify IFN-α6, IFN-α8, and IFN-α14 as the most potent subtypes against HIV-1, markedly suppressing viral replication in humanized mice and ex vivo primary-cell systems, whereas IFN-α1 and IFN-α4 show weak activity. These differences are not absolute: their rank order varies across models, and dose–response dynamics strongly influence outcomes—at high concentrations, subtype disparities diminish, indicating that both intrinsic affinity and ligand exposure jointly shape ISG induction and antiviral efficacy (82–84).

Subtype-specific immune regulation further distinguishes their functional profiles. IFN-α14 and IFN-α8 promote NK- and CD8^+^ T-cell degranulation but induce less inflammatory cytokine release, whereas others preferentially trigger broader ISG and cytokine cascades (84). However, direct evidence that these subtypes cause greater systemic toxicity in patients is currently limited. Most safety data are derived from Peg-IFN-α2–based trials, and whether highly potent subtypes intrinsically confer a poorer tolerability profile remains an open clinical question rather than an established fact.

Despite its moderate antiviral potency, IFN-α2b remains the clinical standard due to its well-established pharmacokinetics, manufacturing stability, and tolerability. Pegylation extends its plasma half-life, smooths exposure peaks, and enables once-weekly administration, providing a controlled immunostimulatory window validated in HBV/HCV and subsequently adapted to HIV clinical research. While next-generation formulations may explore potent subtypes such as IFN-α14, Peg-IFN-α2b currently offers the most reliable balance of antiviral efficacy, safety, and translational feasibility.

### 3.2 Clinical outcomes and stage-dependent effects of IFN-α therapy in HIV-1 infection

Over four decades, multiple clinical and translational studies have evaluated IFN-α as both a stand-alone antiviral and an adjunct to ART in HIV-1 infection. **Table 2** summarizes representative trials spanning the pre-ART, early-ART, and modern-ART eras. These investigations reveal a consistent pattern: short-term IFN-α exposure transiently reduces plasma viremia and integrated HIV-1 DNA, whereas prolonged administration yields diminishing returns accompanied by immune activation and tolerability issues.

**Table 2:** Clinical studies of IFN-α in patients with HIV infection.

Study/Year	ART era	Population (n)	Intervention	Design/Duration	Key findings	Safety/Tolerability	Reference
Frissen et al., 1994	Pre-ART (ZDV monotherapy)	Symptomatic HIV-1 (n = 45)	ZDV ± IFN-α2b 5 MIU s.c. 3×/wk × 48 wk	RCT 48 wk	Combination lowered p24 antigenemia vs ZDV alone; no sustained CD4 gain	Anemia and fatigue (common, reversible)	(85)
Emilie et al., 2001	Early ART (Primoferon A ANRS-086)	Primary HIV-1 (n = 12)	cART + IFN-α 4.5 MIU s.c. 3×/wk × 24 wk	Pilot OBS 24 wk	Early viremia suppression and reservoir decay; enhanced CD8^+^ responses	Mild flu-like symptoms	(86)
Adalid-Peralta et al., 2008	Early ART	Acute HIV-1 (n = 90)	Peg-IFN-α2b 1 µg/kg weekly + ART × 13 wk vs ART alone	RCT 13 wk	IFN enhanced primary antibody responses and transiently reduced VL	Well tolerated (10% grade 1 AEs)	(87)
Asmuth et al., 2010	Transition to modern ART	Chronic HIV-1 (n = 13)	Peg-IFN-α2a 180 µg weekly (monotherapy → add-on ART) × 12 wk	Ph II OL 12 wk	~1 log_10_ VL drop during therapy; immune activation ↑; rebound post-stop	Flu-like AEs; grade 1–2 neutropenia	(26)
Boué et al., 2011 (ANRS-105 INTERVAC)	Modern ART	Chronic HIV-1 (n = 168)	Peg-IFN-α 1.5 µg/kg weekly during structured ATI cycles	RCT multicenter 48 wk	Fewer needed ART restart; no sustained remission after rebound	Fatigue; mild cytopenia (25%)	(88)
Manion et al., 2012	Modern ART	Chronic HIV-1 (n = 10)	Peg-IFN-α2a 180 µg IM weekly × 12 wk	PCS 12 wk	↓ VL; ↑ ISG expression and CD8^+^ activation; no CD4 benefit	Mild cytopenia; reversible	(55)
Azzoni et al., 2013	Modern ART (ATI design)	ART-suppressed (n = 23)	Peg-IFN-α2a 180 µg weekly × 20 wk (add-on) → 4 wk ATI	RCT ATI 24 wk	↓ integrated HIV-1 DNA (0.3 log_10_); 4/23 maintained partial control post-ATI	Grade 2–3 flu-like AEs (25%)	(89)

Pre-ART, period before introduction of combination ART; RCT, randomized controlled trial; s.c, subcutaneous; wk, week(s); PCS, prospective cohort study; OBS, observational study; OL, open-label study; Ph II, phase II clinical trial; VL, viral load; AEs, adverse events; ISG, interferon-stimulated gene; ATI, analytical treatment interruption.

**Table 2** representative clinical studies of IFN-α therapy in HIV-1 infection across pre-ART, early-ART, and modern-ART eras. Short-term IFN-α exposure transiently reduced plasma viremia and integrated HIV-1 DNA, particularly when administered early or as a pegylated add-on to ART. Prolonged treatment yielded limited durable benefit and was associated mainly with mild, reversible flu-like adverse events.

During acute or early infection stages, initial clinical trials have shown that parenteral administration of IFN-α2b can effectively inhibit viral replication before the establishment of viral reservoirs, leading to temporary reductions in viral load and a delayed viral rebound after treatment cessation. However, in chronically infected individuals who have achieved virologic suppression under ART, pegylated IFN-α2a/α2b used as add-on therapy produces only modest reservoir perturbation, including limited reductions in integrated HIV-1 DNA and increased innate and cytotoxic effector activity (26, 54, 89). Importantly, available analytical treatment interruption studies indicate that these changes do not translate into consistent delays in viral rebound or durable post-treatment control, and rebound kinetics correlate more strongly with the size and integrity of the latent reservoir than with prior interferon exposure (89–91). Together, these data indicate that the therapeutic window of IFN-α is phase-dependent: benefits, when present, are most apparent before reservoir consolidation rather than during established chronic infection.

These findings demonstrate that IFN-α can transiently suppress plasma viremia and reduce integrated HIV-1 DNA, especially when used early in infection or as a pegylated adjunct to ART. However, the limited durability of viral control and the frequent occurrence of inflammatory side effects underscore the narrow therapeutic window of IFN-α. The following section examines the immunological consequences of sustained IFN-α signaling, including chronic immune activation, T-cell exhaustion, and their implications for long-term HIV management.

### 3.3 Immunological risks and potential harms of IFN-α in chronic HIV-1 infection

The efficacy of IFN-α in individuals with chronic HIV-1 infection remains controversial. In untreated chronic infection, plasma IFN-α concentrations strongly correlate with plasma HIV-1 RNA levels and markers of immune activation, while inversely correlating with CD4^+^ T-cell counts, suggesting that persistent IFN-α signaling contributes directly to systemic immune activation (39). Mechanistically, IFN-α downregulates IL-7 receptor α (CD127) expression on CD4^+^ and CD8^+^ T cells, in part through sustained JAK–STAT signaling and SOCS induction, thereby impairing IL-7-driven homeostatic proliferation and T-cell recovery (92, 93).

Clinical observations reveal highly variable CD4^+^ T-cell dynamics under IFN-α treatment. In some cohorts, individuals with higher baseline CD4^+^ counts or early-stage disease maintained or exhibited modest, transient increases in CD4^+^ T cells; however, these gains were short-lived (55). The magnitude of CD4^+^ response appears to depend on baseline immune status—patients initiating therapy at higher CD4^+^ levels respond more favorably, whereas those with advanced disease or immune exhaustion show minimal recovery (94, 95). Conversely, individuals with advanced HIV or HIV/HCV co-infection often experience negligible or even declining CD4^+^ counts (96).

The ability of IFN-α to reduce viral load also varies. While some studies have demonstrated reductions in HIV viral load with IFN-α therapy, not all patients respond similarly. Its impact on the viral reservoir appears limited; one study reported poor efficacy in reducing HIV DNA and no significant restriction of the viral reservoir (97). Multiple factors influence treatment outcomes, with a dose-dependent effect observed—higher IFN-α doses are associated with greater reductions in viral load and virological markers but also with increased adverse events and treatment discontinuations (98, 99). Furthermore, the virological and immunological effects of IFN-α are generally transient, with rebound occurring after treatment cessation, and tolerability issues limit long-term use (55, 100).

In summary, the use of IFN-α during chronic HIV-1 infection has not demonstrated sustained clinical benefits and may instead induce or exacerbate chronic immune activation, potentially hindering long-term immune reconstitution. Its application in this setting should therefore be carefully evaluated, taking into account the patient’s immune status, disease stage, and treatment tolerability.

## 4 Emerging strategies and combination immunotherapies

### 4.1 STING agonists

Interferon-based therapy faces a central dilemma: preserving antiviral sensing without provoking systemic inflammation. STING agonists address this by activating cell-intrinsic innate immunity at tissue sites rather than raising circulating interferon. In acute infection, STING activation enhances viral sensing and type I interferon production, limiting early replication. HIV-1 is detected through the cGAS–STING axis, which initiates interferon signaling and antiviral programs (51, 52). In chronic HIV infection, however, plasma viremia is largely controlled by ART, and therapy shifts from suppressing replication to disrupting viral persistence. Accordingly, STING agonists are not intended as direct antivirals. Instead, they are used to perturb the latent reservoir by (i) promoting proviral transcription and chromatin changes that increase “visibility” of infected cells, and (ii) enhancing innate sensing and antigen presentation in lymphoid tissues to facilitate immune clearance. Sustained systemic STING activation is neither necessary nor desirable, as excessive interferon can reproduce immune dysfunction. Current strategies therefore emphasize transient or localized activation to achieve reservoir-directed effects while limiting inflammation. Intercellular transfer of cGAMP can further amplify STING signaling within tissue microenvironments, supporting this localized approach (101).

### 4.2 ncRNA-targeted therapy

The regulatory role of ncRNAs in IFN signaling offers novel therapeutic opportunities. Type I IFN-induced lncRNA ISR2 can enhance antiviral responses when delivered via nanoparticle systems (102), miR-146a—an immunosuppressive miRNA upregulated during HIV infection—can be inhibited by anti-miR-146a, thereby augmenting IFN-mediated T-cell activation (103). Importantly, in this context, “T-cell activation” refers to restoration of antiviral competence (e.g., cytotoxic activity and responsiveness to antigenic stimulation) rather than nonspecific inflammatory activation, which is already excessive in chronic HIV-1 infection. In chronic disease, persistent miR-146a expression may blunt antiviral signaling as part of a negative-feedback program restraining IFN and NF-κB pathways; although anti-inflammatory in principle, this may also contribute to immune dysfunction and incomplete viral control when sustained. ncRNA-based therapy remains conceptual rather than clinical: efficient and cell-specific delivery to CD4^+^ T cells or macrophages is still a major obstacle. Technical barriers include vector safety, off-target effects, and metabolic stability (104, 105).Incorporating ncRNA regulation into IFN-pathway modulation could eventually complement latency-reversing or immune-boosting strategies, but requires significant preclinical validation.

### 4.3 TLR7 agonists

TLR7 agonists, represented by vesatolimod (GS-9620), selectively activate innate immune signaling pathways and induce type I IFN responses. In HIV-infected individuals receiving suppressive ART, vesatolimod has demonstrated a favorable safety profile, eliciting transient increases in ISGs, plasma cytokines, and activation markers on peripheral immune cells without causing sustained inflammation or viral rebound (106). Notably, this contrasts with pegylated IFN-α therapy, in which continuous systemic exposure results in prolonged interferon signaling and cumulative toxicity. In comparison, TLR7 agonists induce short, intermittent interferon “pulses” that more closely resemble physiological innate immune activation rather than sustained pharmacologic exposure.In preclinical nonhuman primate models, TLR7-driven immune stimulation in combination with broadly neutralizing antibodies (bNAbs) significantly delayed viral rebound following ART interruption (107). Parallel studies have shown that combining therapeutic vaccination (Ad26/MVA ± gp140) with intermittent TLR7 administration enhances virologic control and strengthens antiviral T-cell responses, particularly when ART is initiated during acute infection (108). Current translational research focuses on optimizing the timing and sequence of TLR7 agonist administration relative to ART and immunotherapeutic interventions—for instance, priming with bNAbs or therapeutic vaccines before innate immune activation—to maximize efficacy while minimizing immune hyperactivation. In summary, TLR7 agonists targeting the IFN signaling pathway hold considerable promise in HIV cure research and warrant further mechanistic refinement and preclinical evaluation.

## 5 Conclusion

Interferons represent a pivotal bridge between innate and adaptive immunity, exerting potent antiviral and immunomodulatory effects. Among them, type I IFN-α remains the most extensively characterized and therapeutically explored in HIV-1 infection. Evidence from animal models and human trials indicates that early IFN-α administration can restrict viral replication and reservoir establishment. However, during chronic infection, prolonged IFN-α signaling fuels immune activation and T-cell exhaustion. Future approaches must adopt decoupling as a governing principle—preserving antiviral efficacy while disengaging pathways that fuel pathological immune activation.

The clinical limitations of IFN-α therapy in established infection reflect several fundamental barriers, including cumulative toxicity from sustained exposure, reinforcement of immune exhaustion, failure to eradicate the latent reservoir, and viral antagonism of interferon signaling. Collectively, these mechanisms explain why robust induction of interferon-stimulated genes has not translated into durable control *in vivo*. Importantly, these constraints do not negate the therapeutic potential of interferons but redefine the context in which they may be effective.

Recent advances indicate that interferon activity can be repositioned rather than abandoned. Therapeutic strategies that confine interferon exposure to the acute phase of HIV infection, prior to reservoir establishment, may exploit antiviral function when it is biologically most effective and least immunopathogenic. In established infection, interventions that primarily target innate pathways—such as STING agonists, TLR7 agonists, and selected ncRNA-based strategies—are being developed not simply to further amplify cytokine output, but to better control the spatial and temporal dimensions of interferon signaling. By concentrating antiviral signals in relevant tissues and limiting their duration, these approaches aim to reshape the local immune microenvironment while reducing the risk of chronic systemic activation. In this sense, conditioning innate immunity may provide an important foundation for rational combination regimens and enhance the effectiveness of downstream adaptive interventions.

Interferons in HIV should no longer be regarded merely as antiviral cytokines, but as master regulators of immune organization. The future of HIV cure research depends less on intensifying interferon exposure than on programming its deployment—transforming a double-edged pathway into a calibrated therapeutic tool capable of durable impact.
